# Supplementary material for: ERCC1 Overexpression Increases Radioresistance in Colorectal Cancer Cells
Source: Cancers (Basel). 2022 Sep 30;14(19):4798. doi: 10.3390/cancers14194798 (PMC9563575; doi:10.3390/cancers14194798)
Supplement: Supplementary file 1 [file cancers-14-04798-s001.zip › cancers-1835261-supplementary.pdf]

cancers

MDPI

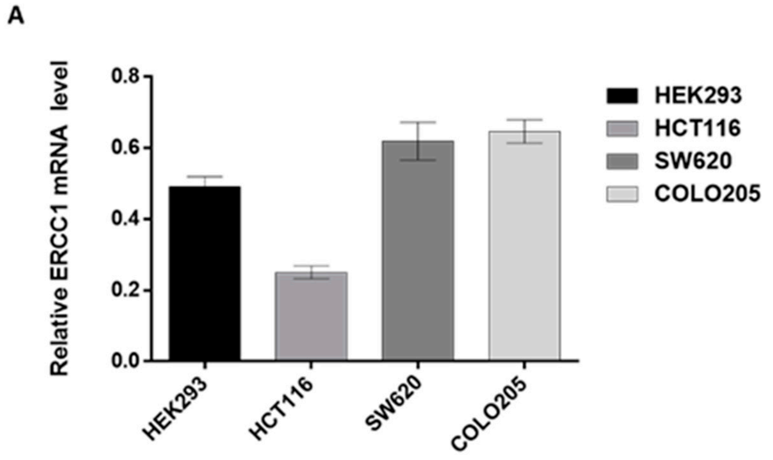

Supplementary Figure S1. Characterization of endogenous ERCC1 level in various cell lines.

To seed 2*10^5/well various cell lines overnight and collected cell mass. Intracellular mRNA was extracted and reverse transcribed into cDNA. *Ercc1* mRNA and beta-actin mRNA were quantified by qPCR. Relative ercc1 mRNA level = ercc1 mRNA level / beta-actin mRNA level. (A) ercc1 mRNA expression in HEK293, HCT116, SW620, and COLO205 cell lines to collect cytoplasmic blocks and analyze endogenous ercc1 and beta-actin expression in cells by western blotting. (B) endogenous ERCC1 level.

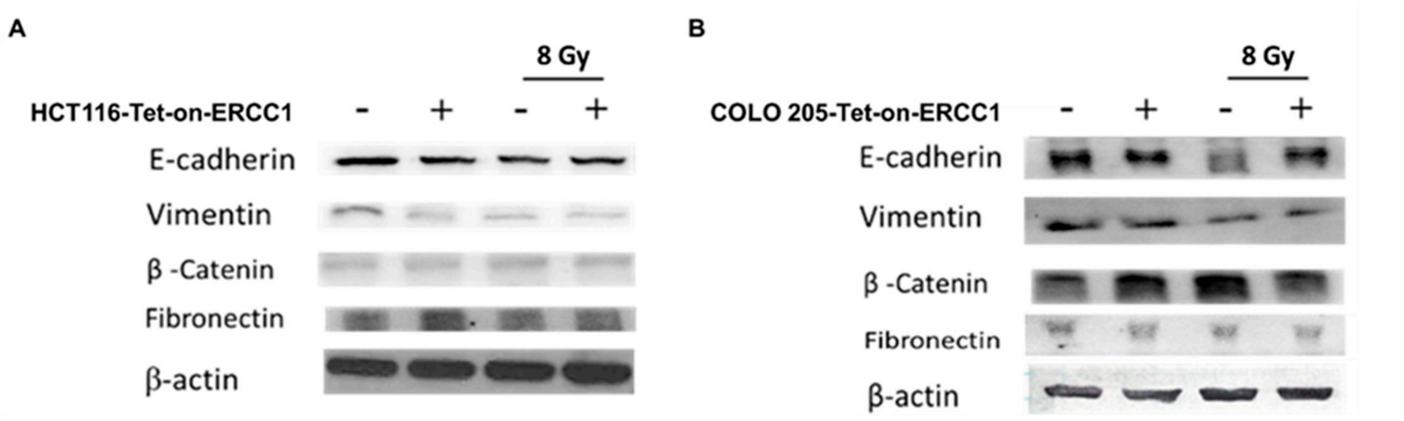

Supplementary Figure S2. Overexpress ERCC1 affects level of EMT markers in colorectal cancer cells.

HCT116-Tet-on-ERCC1 and COLO 205-Tet-on-ERCC1 was treated with 2 μg/ml doxycycline or without after 8Gy exposure or not. Cell pellets were collected and cells were detected for E-cadherin, Vimentin, β-Catenin, Fibronectin, and β-actin by monoclonal antibodies in HCT116-Tet-on-ERCC1 (A) and COLO 205-Tet-on-ERCC1 (B).

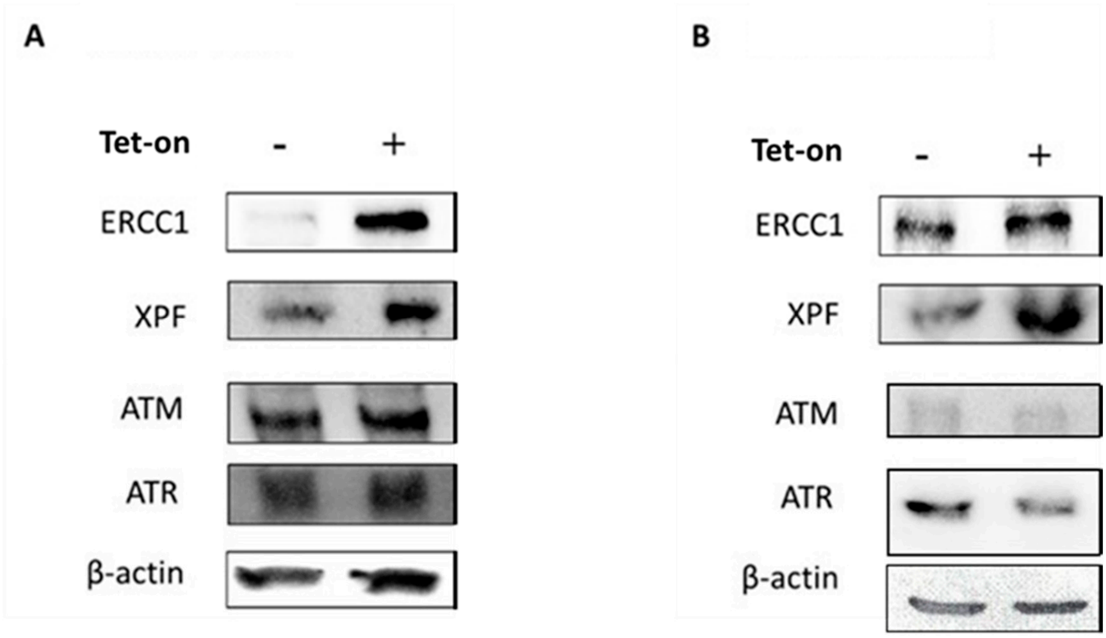

Supplementary Figure S3. Overexpress ERCC1 affects DNA repair factors in colorectal cancer cells.

HCT116-Tet-on-ERCC1(A) and COLO 205-Tet-on-ERCC1(B) was treated with 2 μg/ml doxycycline or without. Cell pellets were collected and cells were detected for ERCC1, XPF, ATM, ATR, and β-actin by monoclonal antibodies.
